# Overexpression of TACE and TIMP3 mRNA in head and neck cancer: association with tumour development and progression

**DOI:** 10.1038/sj.bjc.6606017

**Published:** 2010-11-23

**Authors:** J-W Kornfeld, S Meder, M Wohlberg, R E Friedrich, T Rau, L Riethdorf, T Löning, K Pantel, S Riethdorf

**Affiliations:** 1 Institute of Tumor Biology, Center of Experimental Medicine, University Medical Center Hamburg-Eppendorf, Martinistrasse 52, 20246 Hamburg, Germany; 2 Department of Oral and Maxillofacial Surgery, University Medical Center Hamburg-Eppendorf, Martinistrasse 52, 20246 Hamburg, Germany; 3 Institute of Experimental and Clinical Pharmacology and Toxicology, University Medical Center Hamburg-Eppendorf, Martinistrasse 52, 20246 Hamburg, Germany; 4 Practice of Pathology, Grandweg 64, 22529 Hamburg, Germany; 5 Institute of Pathology, Albertinen-Krankenhaus, Fangdieckstrasse 75a, 22547 Hamburg, Germany

**Keywords:** TACE, TIMP3, squamous cell carcinoma of the head and neck (HNSCC)

## Abstract

**Background:**

TACE/ADAM17 is a transmembranous protease that cleaves membrane-bound growth factors like EGFR ligands. TACE-dependent proteolysis is regulated by its inhibitor, tissue inhibitor of metalloproteinases 3 (TIMP3). This study analyses the role of TACE and TIMP3 mRNA expression in squamous cell carcinomas of the head and neck (HNSCCs).

**Methods:**

We analysed TACE and TIMP3 mRNA expression in HNSCCs from 106 patients by RNA *in situ* hybridisation.

**Results:**

TACE mRNA was upregulated in HNSCCs compared with dysplastic (*P*<0.05) and normal epithelia (*P*<0.001), with strong hybridisation signals in 21.9% of invasive tumour tissues and 4.5% of dysplasia. Elevated mRNA levels were accompanied by increased amounts of TACE protein in HNSCCs. TIMP3 mRNA expression in HNSCC-associated stroma was significantly higher than in the stroma adjacent to dysplastic or normal epithelia. Expression of TACE mRNA in HNSCCs was associated with tumour stage (*P*=0.019) and regional lymph node metastasis (*P*=0.009). Furthermore, levels of TACE mRNA in HNSCCs correlated with the expression of TIMP3 mRNA in HNSCC-associated stroma. Concomitantly, patients expressing high levels of TACE and TIMP3 mRNA showed significantly reduced overall survival compared with those with low mRNA levels.

**Conclusion:**

Our results indicate an important role of TACE and TIMP3 during development and progression of HNSCCs.

Squamous cell carcinoma of the head and neck (HNSCC) is the fifth most common cancer worldwide (Wong *et al*, 1996). Treatment consists of surgery and/or radiotherapy and increasingly chemoradiation, and is associated with reduced speech, swallowing and quality of life. Unfortunately, the 5-year survival rates have not significantly improved in the last two decades and are still ∼50%. The poor prognosis, among others, reflects the limited understanding of the (molecular) mechanisms of HNSCC carcinogenesis itself as well as the lack of reliable HNSCC biomarkers. In many affected patients, recurrences can be found locally or regionally – associated with a high rate of mortality (Woolgar, 2006). Lymph node metastasis strongly correlates with tumour recurrence (Shah, 1990), and treatment failure is often related to growth of disseminated tumour cells either locoregionally causing locoregional recurrences or in lymph nodes outside the original field of treatment (Pantel and Brakenhoff, 2004; Woolgar, 2006).

Invasive growth of tumours is dependent on the degradation of surrounding extracellular matrix (ECM), reflecting dysregulated equilibrium between expression, activation, inhibition and recycling of proteolytic enzymes. The ADAMs (A Disintegrin And Metalloproteinase) are members of the metzincin (zinc-dependent metalloproteases) superfamily (Kheradmand and Werb, 2002) and are structurally highly related to matrix metalloproteinases (MMPs). Currently, >30 ADAM proteins have been characterised, exhibiting functions in fertilisation, neurogenesis and cancer. The ADAMs are characterised by a typical multi-domain architecture (reviewed in Primakoff and Myles, 2000), and some ADAMs exhibit ‘sheddase’ activity; that is, membrane-tethered precursors of cytokines, adhesion molecules and growth factors may be proteolytically ‘shed’ into the extracellular environment. Although an association of MMP expression with tumour cell invasion and metastasis has been demonstrated for a number of malignancies (reviewed in Egeblad and Werb, 2002), there is also increasing evidence for a causal role of ADAM proteins in carcinogenesis and tumour progression (Duffy *et al*, 2009).

The putative role of TACE (tumour necrosis factor (TNF)-*α* converting enzyme), an enzymatically active member of the ADAM sheddase family (ADAM17), during carcinogenesis has been attributed to its ability to shed membrane-tethered molecules crucial for tumour initiation and progression such as TNF-*α* (Black *et al*, 1997), L-selectin (Peschon *et al*, 1998), ALCAM (Rosso *et al*, 2007) transforming growth factor (TGF)-*α* (Kenny and Bissell, 2007) or type I TGF-*β* receptor (Liu *et al*, 2009). Clinically, a potential role for TACE has been proposed for breast, colon, prostate, hepatocellular and ovarian or endocrine cancers (Borrell-Pages *et al*, 2003; Karan *et al*, 2003; Ding *et al*, 2004; Blanchot-Jossic *et al*, 2005; Tanaka *et al*, 2005; Kirkegaard *et al*, 2008; McGowan *et al*, 2008). Intriguingly, TACE possesses a broad substrate spectrum of growth factor and cytokine substrates, whose cleavage leads to activation of the epidermal growth factor receptor (EGFR) signalling cascade (e.g., TGF-*α* and amphiregulin) (Yarden, 2001; Sunnarborg *et al*, 2002; Wang *et al*, 2009). Furthermore, TACE activity also links extracellular G-protein-coupled receptor (GPCR) activation to intracellular EGFR tyrosine phosphorylation (Gschwind *et al*, 2003). Very recent results indicate that TACE regulates EGFR expression through the activation of Notch-1 in non-small cell lung cancer (Baumgart *et al*, 2010). Collectively, an upregulation of TACE-mediated sheddase activity would thus lead to elevated EGFR-derived oncogenic drive in cancer cells, as it is widely accepted that triggering the EGFR downstream cascade is crucial for carcinogenesis (Yarden, 2001; Ford and Grandis, 2003). This holds true especially for HNSCCs (Ang *et al*, 2002). Physiologically, the proteolytic activity of ADAM family members is mainly regulated by its physiological inhibitor, the tissue inhibitor of metalloproteinases 3 (TIMP3) (Amour *et al*, 1998; Baker *et al*, 2002; Walker and Rosenberg, 2009).

The aim of this study was to analyse the expression of TACE mRNA in a series of 106 HNSCC specimens by RNA *in situ* hybridisation. This is the first study relating TACE mRNA expression in HNSCC to the amount of mRNA specific for TIMP3, the primary inhibitor of TACE protease activity *in vitro* and *in vivo*. Our results show that TACE mRNA is upregulated in the majority of human HNSCCs in comparison with their parental epithelia. Furthermore, high levels of TACE mRNA are associated with an increased likelihood of metastasis into regional lymph nodes, high tumour stage, strong stromal TIMP3 expression and reduced overall survival of HNSCC patients.

## Materials and methods

### Patients

Paraffin-embedded HNSCCs from 106 consecutive patients were selected following histological review from the files of the Department of Oral Pathology, University Medical Center Hamburg-Eppendorf, Germany. This study was approved by the local ethics committee. All lesions collected between 1997 and 2004 were classified according to the most recent WHO criteria. Tumours represent primary tumours and originated predominantly from the floor of mouth (30.2%), tongue (35.1%), buccal mucosa (6.8%), upper or lower jaw (6.8%) or the palate (12.2%). Remaining tumours were localised at maxillary or mandibular gingivae, lips or in the oropharynx. The control group consisted of 10 tissue specimens from patients with non-neoplastic, inflammatory disorders of the oral cavity. In addition, snap-frozen human HNSCC samples and adjacent normal tissues were available for immunoblot analysis. Follow-up data were available from 95 patients. The maximal duration of monitoring was 10 years. Median observation time was 29 months. Out of 95 patients, 29 (30.5%) succumbed to the disease. The median survival time of deceased patients was 11 months.

### Cell lines and cell culture

The HNSCC cell line, JCRB 1027 SAT, was obtained from the Japan Health Science Foundation, Health Science Research Resources Bank. The other HNSCC cell lines were kindly provided by Professor RH Brakenhoff (Department of Otolaryngology/Head-Neck Surgery, VUMC Amsterdam, The Netherlands) (Carey *et al*, 1990; Hermsen *et al*, 1996; de Nooij-van Dalen *et al*, 2003; van Zeeburg *et al*, 2005). Cells were grown as monolayers in DMEM medium (Invitrogen, Karlsruhe, Germany) supplemented with 10% fetal calf serum (PAA Laboratories GmbH, Pasching, Austria) and 2 mM L-glutamine (Invitrogen) at 37 °C in a humidified 10% CO_2_ atmosphere.

### Antibodies

The goat polyclonal anti-human antibody sc-6416 (Santa Cruz Biotechnology, Heidelberg, Germany) was raised against the C-terminus of human TACE. The monoclonal mouse anti-human EGFR antibody, clone E30, was raised against the purified, denatured EGFR (Dako Cytomation, Glostrup, Denmark). Mouse monoclonal antibody sc-7298 (Santa Cruz) was raised against the C-terminus of human heat shock cognate protein 70 (HSC-70). Horseradish-peroxidase (HRP) coupled goat-anti-mouse and rabbit-anti-goat antibodies (Dako Cytomation) were used as secondary antibodies.

### Generation of digoxigenin-labelled cRNA probes

Total RNA was extracted from the human HNSCC cell lines VUSCC-OE and UMSCC-14C using Trizol reagent (Invitrogen) and transcribed into cDNA using oligo-dT-primed RT–PCR (Invitrogen).

The following primers corresponding to nucleotides 2748–2772 and 3473-3494 of TACE mRNA (GenBank: NM_003183.4) were used to amplify a 746-bp fragment: forward: 5′-GGGAAGTGACTTAGCAGATGCTGG-3′, reverse: 5′-TCCAAGTGCTGGGATTACAGGC-3′.

To amplify a 445-bp fragment, the following primers corresponding to nucleotides 1448–1472 and 1870–1893 of human TIMP3 mRNA (GenBank: NM_000362.4) were used: forward: 5′-GAGAGTCTCTGTGGCCTTAAGCTGG-3′ reverse: 5′-CTGGGAAGAGTTAGTGTCCAAGGG-3′.

PCR conditions were as follows: initial denaturation for 4 min at 94 °C, followed by 34 cycles of 94 °C for 1 min (denaturation), 64 °C for 1 min (annealing) and 72 °C for 1 min (elongation). The PCR product was analysed on a 1% agarose gel; desired bands were excised and purified using the QIAquick Gel Extraction Kit protocol (Qiagen, Hilden, Germany). The isolated DNA was cloned into a pCRII-TOPO vector (Invitrogen) following the vendor's instructions and the correctness of the insert was confirmed by sequencing. The vector was linearised using *Bam*HI or *Xho*I restriction digest to yield anti-sense or sense probes, respectively.

To obtain cRNA probes from each strand of the cloned sequence, T7 or SP6 polymerases were used. cRNA probes were labelled with digoxigenin using a digoxigenin RNA labelling kit (Roche, Mannheim, Germany) according to the manufacturer's information.

Probes were hydrolysed under alkaline conditions to facilitate probe penetration into tissue. To determine the incubation time necessary for cleavages resulting in a desired length of ∼200 bp, the following calculation was carried out: *t*=(Li–Lf)/(*k* × Li × Lf), with *t*=time in min, Li=initial length in Kb, Lf=final length of ∼200 bp, and *k*=constant 0.11 cuts Kb^–1^ min^–1^. For hydrolysis, the probe was suspended in 100 *μ*l of dH_2_O and 60 *μ*l of 0.2 M Na_2_CO_3_ and 40 *μ*l of 0.2 M NaHCO_3_ were added before incubation at 60 °C for the time span calculated. After stopping the reaction by adding 10 *μ*l of 10% glacial acetic acid and 22 *μ*l of 3 M NaAc, pH 6.0, RNA was precipitated by adding 2 *μ*l of tRNA, 2.4 *μ*l of 1 M MgCl_2_ and 600 *μ*l of ice-cold ethanol. Finally, labelled probes were dissolved in 100 *μ*l Aqua bidest and the digoxigenin-labelling efficiency was determined by dot blotting.

### *In situ* hybridisation

Formalin-fixed paraffin-embedded tissue sections were deparaffinised in *p*-xylene and subsequent washes in graded ethanol. All solutions were prepared with diethylpyrocarbonate (DEPC)-treated H_2_O to inactivate RNAse activity and prevent RNA degradation. To facilitate accessibility of target mRNA, two initial steps using 0.2 N HCl for 20 min and 0.3% Triton X-100 in PBS for 15 min were performed. Target mRNA-bound nucleoproteins were removed by proteinase K incubation (5 *μ*g ml^–1^ for 30 min at 37 °C). Subsequently, the proteinase K activity was quenched via treatment with 0.2% (w/v) glycine for 5 min at 4 °C. Incubation in 4% ice-cold paraformaldehyde (w/v in PBS) ensured proper fixation of tissue onto slides. Acetylation (5 *μ*l ml^–1^ acetic anhydride in 0.1 M triethanoleamine) reduced unspecific background signals. Before hybridisation, slides were dried at 50 °C for 1 h. In an initial step (prehybridisation), the slides were immersed in hybridisation buffer (50% deionised formamide; 1 × Denhardt's solution; 100 *μ*g ml^–1^ Poly-A solution; 500 *μ*g ml^–1^ salmon sperm DNA; 500 *μ*g ml^–1^ t-RNA) for 2.5 h at 52 °C to block unspecific binding. Hybridisation was performed for at least 6 h at 52 °C in hybridisation buffer containing 10% dextran sulphate with a final amount of 50 ng cRNA-DIG probe per slide. Washes with increasing stringency were performed (2 × SSC at RT for 15 min; 1 × in formamide solution (50% Tris-buffered formamide containing 0.3 M NaCl, 1 mM EDTA and 10 mM DTT) at 60 °C for 10 min; 2 × SSC at RT for 3 min; 0.1 × SSC at 52 °C for 15 min; 0.1 × SSC at RT for 10 min). To further remove unbound cRNA probe, the tissue was subjected to RNAse A (0.5 *μ*l ml^–1^) digestion for 30 min. Specifically bound cRNA probe was detected using an anti-DIG-antibody coupled to alkaline phosphatase with nitroblue tetrazolium chloride/X-phosphate 5-bromo-4-chloro-3-indolyl-phosphate (NBT/BCIP) (Roche Diagnostics, Mannheim, Germany) as chromogenic substrate. After an appropriate incubation time the reaction was stopped and sections were counterstained with 1% Nuclear Fast Red dye (Vector, Burlingame, CA, USA), dehydrated using an ascending ethanol concentration chain and *p*-xylene and finally mounted using Entellan mounting media (Merck, Darmstadt, Germany).

### Semiquantitative analysis of *in situ* hybridisation signals

*In situ* hybridisation signals were evaluated using a Zeiss Axioplan 2 light microscope (Göttingen, Germany). Slides were assessed by a board-reviewed pathologist (LR) and two investigators (JWK and SR) independently and blinded to the clinical details of the case. Questionable results were evaluated again until consent was reached. The intensity of TACE/TIMP3 *in situ* hybridisation scores in the tumour was determined semiquantitatively according to the following criteria: TACE/TIMP3^−^=no signal, TACE/TIMP3^+^=weak, TACE/TIMP3^++^=moderate and TACE/TIMP3^+++^=strong. Furthermore, the frequency of positive cells was determined. Histologically discriminable epithelia like normal epithelium (NE), dysplastic epithelium (DE) and stromal areas (STR) adjacent to the invasive tumour were similarly quantified. For subsequent survival analysis and correlation with clinicopathological parameters, *in situ* hybridisation scores in HNSCCs were grouped into only two categories: TACE^lo^, comprising TACE^−^ to TACE^++^ mRNA signals; and TACE^hi^, only TACE^+++^ signals in at least 30% of tumour cells. The intensity of TIMP3 mRNA in stromal tissues adjacent to the tumour was also grouped into two categories: TIMP3^lo^, no signals or signals of weak intensity in stromal cells; and TIMP^hi^, moderate to strong TIMP3 hybridisation signals of stromal cells.

### Immunohistochemistry

After deparaffinisation, tissue sections were pretreated with proteinase K (DAKO) and subsequently endogenous peroxidase was blocked in 0.03% hydrogen peroxide. Anti-EGFR antibody was applied (1 : 100 dilution) overnight at 4 °C. Immunostaining was developed using the peroxidase-labelled polymer and 3,3′-diaminobenzidine (DAB) substrate-chromogen solution. Nuclei were counterstained with haemalaun.

### Immunoblot analysis

The detailed methodology is described in Supplementary Materials and Methods.

### Statistical analysis

Statistical analysis was performed using SPSS for Windows, Version 18.0 (SPSS Inc., Chicago, IL, USA). To test the significance of differences among groups of clinicopathological parameters, data were analysed using two-sided Fisher's exact or *χ*^2^ test. Upregulation of TACE or TIMP3 mRNA *in situ* hybridisation score was shown using the two-sided sign test. The significance level was defined as *P*<0.05. Kaplan–Meier survival analysis of uncensored cases was performed using SPSS.

## Results

### Expression of TACE mRNA

In a hypothesis-driven approach, we determined the expression levels of TACE and TIMP3 mRNA by *in situ* hybridisation in a training set comprising a series of commercially supplied histopathologically defined HNSCC specimens (HNSCC tissue micro array; US Biomax, Inc., Rockville, MD, USA). Interestingly, the intensity of TACE or TIMP3 mRNA tended to be associated with higher tumour stages (T-status) in this group of 57 evaluable carcinomas (*P*=0.067 or 0.09, respectively; Supplementary Table S1). Prompted by this, we aimed to corroborate our findings in a defined set of archived paraffin-embedded tissue from a cohort of 106 HNSCC patients.

**Figure 1:**
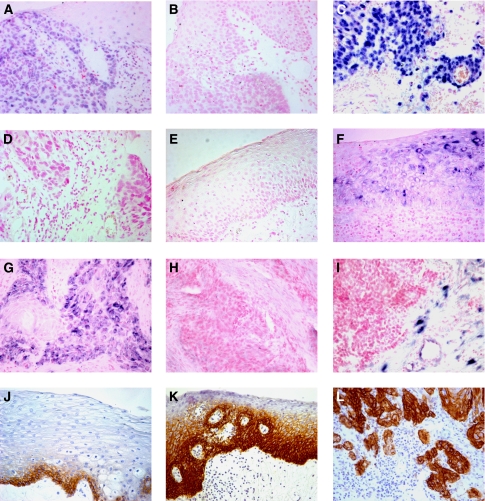
TACE and TIMP3 mRNA expression were detected by RNA *in situ* hybridisation (**A**–**I**, hybridisation signals: blue). (**A**) Moderate TACE mRNA expression in dysplastic epithelium and (**C**) strong TACE hybridisation signals in HNSCC. (**E**) No TACE mRNA expression in normal epithelium. Control riboprobes in sense orientation against TACE ((**B**) control to (**A**), (**D**) control to (**C**), and TIMP3 (**H**) to (**G**)) yielded no signals. Moderate to strong TIMP3 mRNA-specific signals are found in dysplastic epithelium (**F**), in HNSCC (**G**) and stromal areas adjacent to HNSCC (**I**). EGFR protein detected by immunohistochemistry (**J**–**L**). EGFR protein is highly expressed in basal and parabasal cells of normal epithelium (**J**), dysplastic (**K**) and HNSCC (**L**) epithelia. Slides were counterstained with nuclear fast red dye (*in situ* hybridisation, pink) and haematoxylin (immunohistochemistry, blue); original magnification: × 200.

**Figure 2:**
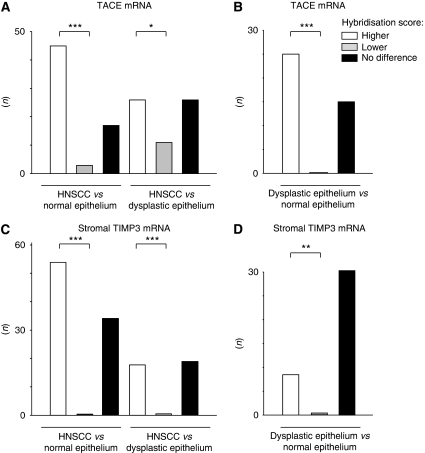
TACE *in situ* hybridisation signals in head and neck tissues and TIMP3 *in situ* hybridisation signals in stromal cells surrounding HNSCC, dysplastic and normal epithelium. *In situ* hybridisation signals of paraffin sections were scored semiquantitatively. TACE mRNA expression levels were compared between tumour and normal epithelium or tumour and dysplastic epithelium (**A**). Dysplastic areas were compared with normal epithelium (**B**). TIMP3 mRNA expression in stromal areas surrounding tumour epithelium were compared with normal or dysplastic epithelium (**C**). Stromal areas surrounding dysplastic areas were compared with normal epithelium (**D**). Statistical significance was calculated using nonparametric sign test. The *P*-values of <0.05 were defined as statistically significant (^*^P<0.05; ^**^P<0.01; ^***^P<0.001).

Hybridisation signals specific for TACE were found throughout the oral mucosa with varying intensities between specimens and tissues (Supplementary Table S2, Figure 1A and C). *In situ* hybridisation with the control sense riboprobe yielded no signals (Figure 1B and D). Signals also disappeared when samples underwent an additional RNAse treatment (not shown). Hybridisation signals specific for TACE were also found in non-epithelial cells like vessel endothelial cells and lymphocytes (not shown). Histologically NE adjacent to HNSCC was either negative for TACE mRNA (71.9%, Figure 1E) or expressed it only weakly (25%) to moderately (3.1%). Samples of patients suffering from non-cancerous, inflammatory diseases of the oral cavity displayed comparable levels of TACE mRNA in NE (not shown). In contrast, only a few cases of DE adjacent to the tumours were negative for TACE mRNA (27.3%); weak (31.8%) to intermediate (36.4%) hybridisation signals were predominant (Figure 1A). Only a small number of patients expressed high levels of TACE mRNA in DE (4.5%). A comparable percentage of HNSCCs was negative for TACE mRNA (19.0%), and similarly the majority of tumours displayed weak (22.9%) to intermediate (36.2%) signals. A considerable number of tumours, however, exhibited high amounts of TACE mRNA (21.9%, Figure 1C), thus clearly differing in signal intensity from dysplastic areas (Figure 1A). Stroma associated with HNSCC was predominantly negative for TACE-specific hybridisation signals (74%, Supplementary Table S2) and showed, if any, weak (23%) or moderate (3%) amounts of TACE mRNA. The upregulation of TACE mRNA in HNSCC compared with NE (*P*<0.001, Figure 2A) and DE (*P*<0.05, Figure 2A) was statistically significant. Finally, DE exhibited elevated levels of TACE mRNA compared with NE (*P*<0.001, Figure 2B).

Although moderate and high EGFR protein levels were detected in most HNSCC cases (data not shown in detail, representative image in Figure 1L), a strong increase of EGFR protein expression from NE to DE and carcinoma tissue was observed (Figure 1J–L).

### Relationship between TACE mRNA expression and clinicopathological parameters

To assess whether high levels of TACE mRNA correlate with HNSCC progression, we defined TACE^lo^ and TACE^hi^ carcinomas and juxtaposed levels of TACE mRNA with known clinicopathological parameters (Table 1). The nodal status was assessed in 91 patients, 30 of whom had lymph node metastases. We found no statistical association of TACE mRNA levels with age or gender of affected patients. Interestingly, 36.7% of lymph node-positive (LN^+^) patients displayed high levels of TACE mRNA (TACE^hi^). In contrast, only 13.1% of lymph node-negative (LN^−^) patients were scored as TACE^hi^. The correlation between the amount of TACE mRNA and the presence of metastases in regional lymph nodes was statistically significant (*P*=0.009). The positive correlation of TACE mRNA levels with primary tumour stage was also statistically significant (*P*=0.019).

### Expression of TACE protein

**Figure 3:**
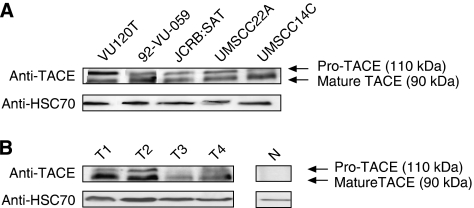
Representative western blot showing TACE protein expression in (**A**) HNSCC cell lines and (**B**) human HNSCC (T1–4) and adjacent normal tissue (N). Two distinct bands constitute unprocessed and cleaved mature TACE protein. Blotting results obtained with an anti-HSC70 antibody were used as loading control.

To elucidate whether TACE mRNA overexpression in human HNSCC results in corresponding levels of translated TACE protein, we conducted immunoblot analysis using an antibody specifically recognising the carboxyterminal part of human TACE. To verify antibody specificity, we analysed a panel of well-characterised HNSCC cell lines for the expression of TACE protein. We found two clearly discriminable bands migrating with an approximate molecular mass of 110 and 90 kD, respectively. The ratio between cleaved and uncleaved TACE protein varied between different HNSCC cell lines (Figure 3A). Based on the molecular mass, these bands constitute the uncleaved precursor as well as the enzymatically active mature TACE protein. The observed molecular weight of TACE protein was in line with most other reports utilising TACE-specific polyclonal antibodies (Blanchot-Jossic *et al*, 2005; McGowan *et al*, 2007). To find out whether TACE protein could also be detected in carcinoma tissue, we analysed tissue lysates from four human HNSCC specimens. Immunoblot showed heterogeneous TACE protein expression exclusively in tumour but not in normal oral tissues (Figure 3B). In HNSCC, the mature TACE isoform constituted the dominant isoform.

### Expression of TIMP3 mRNA

Tissue inhibitor of metalloproteinases 3 mRNA was detected throughout all oral tissues analysed (NE, DE and cancerous lesions; Supplementary Table S3). Histologically NEs harboured low levels or intermediate levels of TIMP3 mRNA; the same held true for DEs (Figure 1F) and the majority of cancerous lesions (Figure 1G, Supplementary Table S3). Yet, no statistically significant upregulation of TIMP3 mRNA levels could be found between these epithelial areas. One hallmark of TIMP3 biology is the fact that this cell-secreted soluble molecule can be expressed either by tumour cells at the invasion front or in the surrounding stroma. Thus, TIMP3 mRNA levels were markedly increased in stromal areas surrounding invasive HNSCC compared with DE and NE (Figures 1I and 2C and Supplementary Table S3) and were also elevated in the stroma adjacent to DE compared with NE (Figure 2D and Supplementary Table S3).

### Relationship between TIMP3 mRNA expression and clinicopathological features

Expression levels of TIMP3 mRNA in the tumour tissues did not correlate with clinicopathological parameters of the patients analysed. However, considering tumour-associated stromal areas separately resulted in a significant correlation of increased TIMP3 mRNA expression with a higher tumour stage (*P*=0.031, Table 1). Furthermore, a significant correlation between levels of TIMP3 mRNA in tumour-associated stromal cells and TACE mRNA in tumour cells was detected (*P*=0.022, Table 1).

### Impact of TACE and TIMP3 mRNA overexpression on overall survival of patients

**Figure 4:**
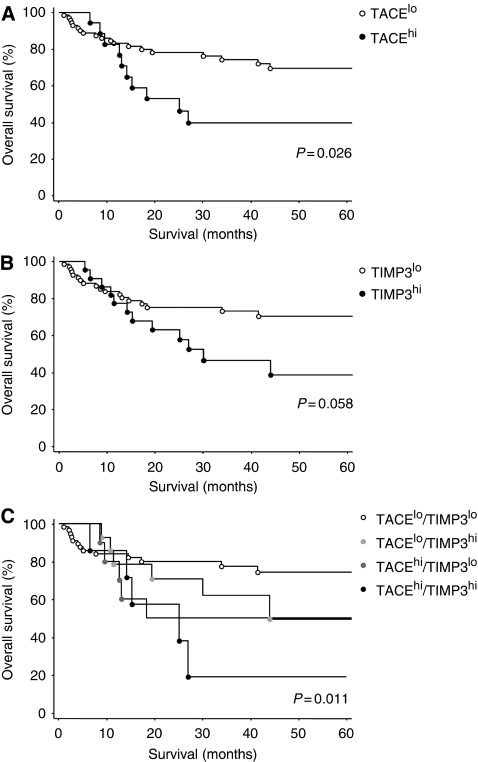
Kaplan–Meier survival curve demonstrating percentage of overall survival in patients whose tumours harboured high *vs* low levels of TACE mRNA in tumour cells (**A**) and TIMP3 mRNA in stromal cells (**B**). Overall survival of patients with high TACE and TIMP3 mRNA levels, high TACE or high TIMP3 mRNA levels or low TACE and low TIMP3 mRNA levels in tumour and stromal cells, respectively (**C**).

Finally, we aimed to investigate a possible correlation between TACE and TIMP3 mRNA expression levels and the overall survival of patients. Of 95 affected patients with known follow-up data, those individuals whose tumours showed TACE^hi^ mRNA expression levels exhibited reduced overall survival compared with those with TACE^lo^ mRNA expression, as judged by Kaplan–Meier analysis. This unfavourable prognosis proved to be statistically significant (*P*=0.026, log-rank test, Figure 4A). However, we could not show that this influence on survival was independent of lymph node metastasis (data not shown). There was also a correlation of borderline significance between high TIMP3 mRNA levels in tumour-associated stromal areas and a reduced overall survival of HNSCC patients analysed (*P*=0.058, Figure 4B). Combining the results of TACE and TIMP3 mRNA detection, patients with both high levels of TACE and TIMP3 mRNA had the worst clinical outcome, followed by patients with either high levels of TACE or TIMP3 mRNA levels. The most favourable overall survival was observed in patients with low levels of TACE mRNA in tumour cells and low levels of TIMP3 mRNA in stromal cells (*P*=0.011, Figure 4C).

## Discussion

We determined the amount of TACE and TIMP3 mRNA in human HNSCC using RNA *in situ* hybridisation as the method of choice, as published reports and own studies quantifying TACE protein levels using immunohistochemistry led to conflicting results, probably because of poorly characterised antibodies (Merchant *et al*, 2008).

The positive correlation between TACE transcript levels and increasing malignancy of HNSCC argued for TACE mRNA upregulation being an important early event in tumourigenesis itself as well as in the transition from premalignant lesions to HNSCC. Bioinformatic screens for differential TACE mRNA expression using the Oncomine database (www.oncomine.org) confirmed the highly significant (*P*<10^−6^) increase in TACE transcript levels in HNSCC specimens (Cromer *et al*, 2004). Furthermore, in our study, TACE mRNA levels correlated with nodal metastases and primary tumour size and concomitantly corroborated a report describing the association between TACE protein levels, nodal metastasis and local recurrences in a small HNSCC cohort (Takamune *et al*, 2007). Recently, close correlations between TACE sheddase activity and HNSCC tumour stage were reported (Ge *et al*, 2009). Intriguingly, in our patient cohort, elevated TACE mRNA levels in HNSCC correlated with reduced patient survival. This may be related directly to the observation that TACE mRNA levels parallel the degree of lymph node metastasis, an independent prognostic marker for tumour progression and patient survival.

We analysed the expression of TACE protein in human HNSCC cell lines and HNSCC tumour samples and detected high levels of both mature and immature TACE protein. In tumour tissue we found a prevalence of the lower molecular weight, enzymatically active TACE isoform. Further studies need to confirm the correlation between TACE mRNA and enzymatically active TACE protein levels. Conflicting migratory patterns of TACE proteins in published immunoblot analyses (Takamune *et al*, 2007) might be because of different antibodies used.

Thus, TACE represents one key regulator of EGFR ligand availability (Sunnarborg *et al*, 2002) and bioactivity (Borrell-Pages *et al*, 2003; Lemjabbar *et al*, 2003). Epidermal growth factor receptor is widely accepted as a crucial signalling platform on which growth factor signals converge, and the effect of its overexpression in HNSCC tumours has been described extensively (Ford and Grandis, 2003; Gschwind *et al*, 2003; Fischer *et al*, 2004; Schafer *et al*, 2004). Interestingly, moderate and high levels of EGFR protein were detected in the majority of HNSCCs analysed in this study. Thus, elevated TACE levels might contribute to the bioavailability of EGFR ligands in HNSCC, providing cancer cells with high TACE expression levels with a selection advantage. Kenny and Bissell (2007) showed that inhibition of TACE protease activity blocked EGFR signalling, resulting in a reversion of the malignant phenotype of T4-2 breast cancer cells. Inhibition of TACE using selective inhibitors also decreased cell proliferation in an *in vitro* model of colorectal cancer (Merchant *et al*, 2008).

Tissue inhibitor of metalloproteinase 3 exerts its anti-proteolytic function either at the invasion front of an infiltrating HNSCC to quench tumour-associated ECM degrading activity or in the stroma itself, where soluble proteases liberate ECM-tethered factors that assist the cancer cell in migration and invasion. Proper balancing between proteolytic enzymes (e.g., MMPs) and their cognate inhibitors (e.g., TIMPs) seems to be of importance for cancer metastasis and invasion (Cruz-Munoz *et al*, 2006; Wu *et al*, 2008). Our finding of TIMP3 upregulation in HNSCC with increasing tumour stage is in contrast to a published report that claimed a tumour-suppressor function for TIMP3, showing to be frequently silenced by promoter hypermethylation during HNSCC progression (Worsham *et al*, 2006). Comparing expression levels of TIMP3 mRNA in TACE^lo^ and TACE^hi^ tumours, we observed a positive correlation between levels of TIMP3 in tumour-associated stromal tissues and TACE mRNA in HNSCC. Furthermore, high expression of both tumour-associated TACE and stromal TIMP3 mRNA led to worse clinical outcomes than either factor independently. Hitherto, no reports linked TACE activity to increased TIMP3 mRNA transcription, and thus this effect probably constitutes a compensatory mechanism to counterbalance the pathological increase in TACE sheddase activity. Yet, it cannot be excluded that TIMP3 expression may also function to promote tumour progression (Byrne *et al*, 1995).

Collectively, this study demonstrates the importance of TACE and TIMP3 expression in HNSCC tumourigenesis and progression. Further investigation is necessary to identify the key proteins shedded by TACE that are pivotal for the development and progression of these tumours.

## Figures and Tables

**Table 1:** Expression of TACE mRNA in HNSCC and TIMP3 mRNA in stromal tissues in relation to clinicopathological parameters

**Clinicopathological feature**	***n***	**TACE^lo^**	**TACE^hi^**	***P-*value**	***n***	**TIMP3STR^lo^**	**TIMP3STR^hi^**	***P-*value**
**Quantification**	**ISH**				**ISH**			
*Age*								
⩽60 years	50	38	12	>0.5	50	35	15	0.312
>60 years	55	44	11		56	44	12	
								
*Sex*								
Male	69	53	16	>0.5	71	51	20	0.364
Female	36	29	7		35	28	7	
								
*Nodal status*								
LN^−^	61	53	8	***0.009***	61	47	14	>0.5
LN^+^	30	19	11		31	22	9	
								
*Tumour stage*								
T1	39	61	12	***0.019***	39	60	13	***0.031***
T2	34				34			
T3	8	18	11		8	18	11	
T4	21				21			
								
*Histological grading*								
G1	13	64	17	>0.5	13	62	19	>0.5
G2	68				68			
G3	24	18	6		24	17	7	
								
*TIMP3 (ISH)*								
TIMP3STR^lo^	77	64	13	***0.022***				
TIMP3STR^hi^	26	16	10					

Abbreviations: HNSCC=squamous cell carcinoma of the head and neck; TACE=tumour necrosis factor (TNF)-*α* converting enzyme; LN=lymph node; TIMP3=tissue inhibitor of metalloproteinases 3; ISH=*in situ* hybridisation. Bold and italic entries indicate statistically significant values.
