# Supplementary material for: Overexpression of TACE and TIMP3 mRNA in head and neck cancer: association with tumour development and progression
Source: Br J Cancer. 2010 Nov 23;104(1):138–45. doi: 10.1038/sj.bjc.6606017 (PMC3039790; doi:10.1038/sj.bjc.6606017)
Supplement: Supplementary Tables [file 6606017x1.doc]

**Supplementary Tables**

**Table S1: Expression of TACE mRNA in HNSCC (TMA HN 802) and TIMP3 mRNA in stromal tissues in relation to clinicopathological parameters**

| **Clinicopathological feature** | **n** | **TACE^lo^** | **TACE^hi^** | **p** | **n** | **TIMP3STR^lo^** | **TIMP3STR^hi^** | **p** |
| --- | --- | --- | --- | --- | --- | --- | --- | --- |
| **Quantification** | *In situ* hybridization, ISH | | | | ISH | | | |
| **Age** |  |  |  |  |  |  |  |  |
| ≤ 60 years | 39 | 36 | 3 | *0.094* | 39 | 33 | 6 | *0.297* |
| > 60 years | 18 | 13 | 5 |  | 18 | 13 | 5 |  |
| **Sex** |  |  |  |  |  |  |  |  |
| Male | 48 | 41 | 7 | *>0.5* | 48 | 38 | 10 | *>0.5* |
| Female | 9 | 8 | 1 |  | 9 | 8 | 1 |  |
| **Nodal Status** |  |  |  |  |  |  |  |  |
| LN^-^ | 41 | 36 | 5 | *>0.5* | 41 | 35 | 6 | *0.26* |
| LN^+^ | 16 | 13 | 3 |  | 16 | 11 | 5 |  |
| **Tumor Stage** |  |  |  |  |  |  |  |  |
| T1 | 11 | 24 | 1 | *0.067* | 11 | 23 | 2 | *0.09* |
| T2 | 14 |  |  |  | 14 |  |  |  |
| T3 | 13 | 25 | 7 |  | 13 | 23 | 9 |  |
| T4 | 19 |  |  |  | 19 |  |  |  |
| **Histological**  **Grading** |  |  |  |  |  |  |  |  |
| G1 | 22 | 42 | 5 | *0.312* | 22 | 37 | 10 | *>0.5* |
| G2 | 25 |  |  |  | 25 |  |  |  |
| G3 | 9 | 7 | 2 |  | 9 | 8 | 1 |  |
| **TIMP3 (*ISH)*** |  |  |  |  |  |  |  |  |
| TIMP3STR^lo^ | 46 | 40 | 6 | *>0.5* |  |  |  |  |
| TIMP3STR^hi^ | 11 | 9 | 2 |  |  |  |  |  |

**Table S2: Expression of TACE mRNA in different oral tissues**

| **Tissue** | TACE mRNA  n (%) | | | |
| --- | --- | --- | --- | --- |
|  | - | + | ++ | +++ |
| **Normal**  **Epithelium (NE)** | 46  (71.9) | 16  (25.0) | 2  (3.1) | 0 |
| **Dysplastic**  **Epithelium (DE)** | 18  (27.3) | 21  (31.8) | 24  (36.4) | 3  (4.5) |
| **HNSCC** | 20  (19.0) | 24  (22.9) | 38  (36.2) | 23  (21.9) |
| **Stroma adjacent**  **to HNSCC** | 74  (74.0) | 23  (23.0) | 3  (3.0) | 0 |

**Table S3: Expression of TIMP3 mRNA in different oral tissues**

| **Tissue** | TIMP3 mRNA  n (%) | | | |
| --- | --- | --- | --- | --- |
|  | - | + | ++ | +++ |
| **Normal**  **Epithelium (NE)** | 78  (87.6) | 10  (11.2) | 1  (1.1) | 0 |
| **Stroma adjacent**  **to NE** | 87  (97.8) | 2  (2.2) | 0 | 0 |
| **Dysplastic**  **Epithelium (DE)** | 24  (66.7) | 9  (25.0) | 3  (8.3) | 0 |
| **Stroma adjacent**  **to DE** | 28  (77.8) | 7  (19.4) | 1  (2.8) | 0 |
| **HNSCC** | 78  (73.6) | 21  (19.8) | 5  (4.7) | 2  (1.9) |
| **Stroma adjacent**  **to HNSCC** | 40  (37.7) | 39  (36.8) | 15  (14.2) | 12  (11.3) |

**Supplementary Materials and Methods:**

Immunoblot analysis

HNSCC cell line cells were lysed in 250-500 µL ADAM lysis buffer (20mM Tris-HCl (pH 7.4), 150 mM NaCl, 0.5 % Triton X-100, 0.1 % SDS, 1 mM EDTA, 1 mM sodium orthovanadate and 10 mM 1,10 o-phenanthroline (to prevent autolysis of ADAM proteins) and 1xProtein Inhibitor Cocktail Set I (Calbiochem/EMD, San Diego, CA, USA)) on ice for 45 min. The cell suspension was centrifuged at 20,000 g at 4 °C for 15 min to remove cell debris. Snap-frozen human head and neck tumor tissues were homogenized by standard procedures, taken up in ADAM lysis buffer and incubated on ice for 45 min. Subsequently, insoluble tissue debris was removed by centrifuging at 20,000 g for 20 min at 4 °C. The supernatant of lysed oral tissue and HNSCC cell lines was used to measure protein concentration using Bradford® reagent (Bio-Rad, Hercules, CA, USA) according to manufacturer’s protocol. Cell lysates were taken up in reducing SDS gel-loading buffer (4 % (w/v) SDS, 20 % glycerol, 120 mM Tris-HCl pH 6.8, 0.01 % brom-phenole-blue and 20 mM DTT). Twenty micrograms of protein were heated at 95 °C for 5 min prior to loading onto a 10 % SDS-PAGE gel. Subsequently, proteins were transferred onto Immobilon-P PVDF membrane (Millipore, Schwalbach, Germany) according to standard Western blotting procedure. The membrane was blocked at RT for 30 min in PBS containing 4 % (w/v) dry milk (blocking buffer) and incubated in a 1:500 dilution of polyclonal goat-anti-human TACE primary antibody in blocking buffer. After washing the membrane thoroughly with PBS containing 0.05 % Tween-20, a 1:5000 dilution of rabbit-anti-goat-HRP in blocking buffer was added. For detection, the membrane was incubated in 8 mL of ECL Detection Reagent (Amersham, Piscataway, NJ, USA) and exposed to X-ray film.
